# Indicators of Household Composition Are Associated with Adherence to Fruit and Vegetable Intake Recommendations Among Caretakers Eligible for SNAP with Children

**DOI:** 10.3390/ijerph23060765

**Published:** 2026-06-06

**Authors:** Kellie McLean, Stefani Wiloejo, Zoya N. Rehman, Pasquale E. Rummo, Angela C. B. Trude

**Affiliations:** 1 Department of Nutrition and Food Studies, Steinhardt School of Culture, Education, and Human Development, New York University, 411 Lafayette Street, 5th Floor, New York, NY 10003, USA; 2 Department of Population Health, Grossman School of Medicine, New York University, 227 East 30th Street, 6th Floor, New York, NY 10016, USA

**Keywords:** fruit and vegetable intake, household, children, food assistance programs, low-income

## Abstract

**Highlights:**

**Public health relevance—How does this work relate to a public health issue?**

- The findings of this study relate to public health as they show that having more children participating in school lunch programs was positively associated with greater caretaker fruit and vegetable consumption.
- These findings may further direct nutrition education programs that aim to lower disease incidence.

**Public health significance—Why is this work of significance to public health?**

- Indicators of increased fruit and vegetable consumption among low-income households, such as having children present, are of significance to public health to assist with the design of educational programs aiming to lower disease incidence.

**Public health implications—What are the key implications or messages for practitioners, policy makers and/or researchers in public health?**

- The key takeaway for public health professionals is that having children in the household in the context of a city that offers universal free meals may be a factor associated with caretakers’ increased fruit and vegetable intake.
- Future studies should be conducted that investigate food behaviors among families of low income to design future educational programs and interventions promoting increased fruit and vegetable intake.

**Abstract:**

Inadequate fruit and vegetable consumption is associated with increased risk of chronic disease. Yet, many individuals consume below the recommended intake according to the Dietary Guidelines for Americans (DGA). This study aimed to examine the association of adherence to the DGA (2020–2025) recommendations for fruit and vegetable (FV) intake of 1.5–2 cups of fruits and 2–3 cups of vegetables daily for adults among caretakers with a child(ren) living in households eligible for a Supplemental Nutrition Assistance Program (SNAP). We conducted a cross-sectional analysis of 85 caretakers with children in an urban neighborhood of low-income in the Bronx, New York (NY). Log-binomial regressions demonstrated that having more children (RR 1.36; 95% CI 1.15–1.59), younger children (RR 1.22; 95% CI 1.07–1.39), or children participating in a school lunch program (RR 1.47; 95% CI 1.16–1.85) was positively associated with caretakers’ probability of adhering to the DGA recommendations for FV intake. Our study highlights the eating behaviors of families living with children ≤ 10 years of age, many of whom were participating in a school lunch program, and underscores the dietary benefits associated with these characteristics.

## 1. Introduction

Adequate intake of fruits and vegetables (FVs) is associated with a reduced risk for many chronic diseases, such as obesity [1,2], type 2 diabetes mellitus [3,4], cardiovascular disease [5], and cancer [5,6,7]. The Dietary Guidelines for Americans (DGA) 2020–2025 recommends that adults in the United States (U.S.) consume 1.5–2 cups of fruits and 2–3 cups of vegetables daily [8]. However, only 10% of adults meet the fruit and vegetable (FV) intake recommendations [9].

High food costs, limited availability, and lack of access to FVs, particularly in communities of low income who disproportionately consume fewer FVs than their counterparts, are among the multifaceted environmental barriers to healthy eating [8,10]. Concurrently, adults living in households of low income report limited time to shop and cook as an individual-level barrier to FV intake [8]. The household composition may also play a role in FV intake, as it is associated with eating behaviors that influence food allocation and social norms [11]. Having more children in the household may increase food budgets and preparation time, leading to lower FV intake, particularly among caretakers who may modify their diet to provide for their children in the context of food insecurity [12].

Previous studies examining household composition and diet have focused on household food purchases [12,13,14,15,16], intake [17], or the children’s diet [17,18,19,20]. These studies have yielded mixed results. Some reported that having more children in the household predicted higher fruit intake in children [20], whereas others showed a negative association between the number of children and their FV intake [17,18] as well as vegetable variety [18]. Nonetheless, few studies consider the role of household composition in adult caretakers’ FV intake [21,22,23,24,25]. It is important to understand how indicators of household composition may be associated with the caretakers’ diet, as they are important role models and may shape children’s food choices over time [26]. Most of the existing literature compared FV intake between parents and non-parents, with results showing that caretakers consume more sugar-sweetened beverages, total calories, and percent saturated fat as well as fewer FVs [21,22,23,24,25] due to having less time to eat healthy and consuming more snacks with their children [21]. However, there may be important heterogeneity aside from the children’s presence within households that warrants investigation regarding caretakers’ FV intake.

Numerous studies have examined children’s characteristics, such as age and participation in a school lunch program, as indicators of household composition [27,28,29,30,31,32]. Two cross-sectional studies [27,28] found that children who are older tend to consume fewer fruits [31] and adopt unhealthier dietary patterns compared to their younger counterparts [32] due to different levels of support from caretakers on FV intake and their emerging independence in food choices [27]. Thus, caretakers with children who are older may purchase and consume fewer FVs. Other studies found that having more children in the household participating in a school lunch program decreased food spending, providing the opportunity to reallocate the food budget to higher-quality food items among other household members [29,30]. Such studies have urged the current body of literature to move beyond the measure of children’s presence in the household to include the number of children and children’s characteristics within the household composition.

Therefore, our study contributes to the current body of literature by considering an array of indicators of household composition, including household size, number of children, children’s age, their participation in a school lunch program, and number of adult caretakers, to examine the association with adherence to DGA recommendations for FV intake among caretakers with a child(ren) living in households eligible for a Supplemental Nutrition Assistance Program (SNAP).

## 2. Materials and Methods

### 2.1. Participants

We conducted a cross-sectional analysis of 85 caretakers eligible for the SNAP with a child(ren) in the Bronx, New York (NY), USA to examine the association between indicators of household composition and adherence to the DGA 2020–2025 recommendations for FV intake among caretakers, defined as biological parents, non-biological parents, or blood relatives, such as grandparents. Our analysis was part of a parent study that examined the feasibility of an online grocery intervention to support healthy food selection among households of low income with children. The New York University Institutional Review Board (IRB-FY2022-6623) approved our study protocol, and all individual participants provided written informed consent virtually.

### 2.2. Data Collection

We collected data between July and September 2022 in five Head Start centers located in two neighborhoods in the Bronx, NYC, with the highest poverty rates compared to the city estimates [33].

We approached caretakers who, if interested, scanned a barcode or texted our study’s texting line to receive a link to an online survey available in English and Spanish via Qualtrics. Caretakers answered screening questions to assess eligibility for the parent study. They were included if they (1) are the primary food shopper of a household with a child(ren) ≤ age 10, (2) have been a SNAP participant in the past year or have an annual household income ≤ 130% of the federal poverty level, (3) have a smartphone to facilitate participation in the parent intervention, and (4) speak English or Spanish. Those who met the eligibility requirements continued to answer other survey questions regarding demographic characteristics (i.e., age, sex, race/ethnicity, and preferred language), household food security via the United States Department of Agriculture (USDA) 6-item Food Security Survey [34], SNAP participation in the past year, indicators of household composition (i.e., household size, number of children ≤ age 10, children ≤ age 5, and children participating in a school lunch program), and FV intake via the National Care Institute (NCI) FV Screener [35]. We used reCAPTCHA verification and cookie technology to confirm human answers and ensure only a single submission was received per participant, respectively. Caretakers took approximately 15 min to complete the online survey and received $20 after completing it.

### 2.3. Measures

#### 2.3.1. Outcome Variable

We assessed the caretakers’ FV intake using the NCI FV Screener. The screener is a self-reported assessment of usual FV intake in either cups or servings. In accordance with the 2005 MyPyramid Dietary Guidance, we defined a serving as 2 cups of raw, leafy vegetables or 1 cup of other vegetables, cooked or chopped raw; 1 cup of juice; one whole fruit; or 1 cup of cut-up fruit [36]. The screener inquired about the amount as well as daily, weekly, and monthly intake of ten FV categories (i.e., juice, fruits, lettuce salad, French fries/fried potatoes, other white potatoes, cooked dried beans, other vegetables, tomato sauce, vegetable soups, and mixtures that included vegetables) in the past month [35]. Some advantages include its validity, high association with 24 h recalls, ease of use, and low cost compared to other measurement tools [35,37,38].

#### 2.3.2. Exposure Variable

Caretakers self-reported their household size via multiple-choice questions (with options ranging between 2 and 10+), number of children ≤ age 10 (between 1 and 6+), number of children ≤ age 5 (between 1 and 5+), and number of children participating in a school lunch program (between 1 and 6+).

We calculated the ratio of adults to children by dividing the number of adults by the number of children ≤ age 10 (adult-children). We obtained the number of adults by subtracting the number of children ≤ age 10 from the household size. Similarly, we calculated the ratio of younger children (≤age 5) to older children (ages 6–10) by dividing the number of younger children by older children (younger–older children). We obtained the number of older children by subtracting the number of younger children from the number of children ≤ age 10. Given that participation in our study required having at least one adult caretaker and one child ≤ 10 years of age, denominator variables were always ≥1.

#### 2.3.3. Covariates

The online survey included multiple-choice questions regarding age (with options ranging between 18–24 and 65+), sex (female, male, non-binary, prefer not to say, or prefer to self-describe), race/ethnicity (African American/Black, American Indian/Alaska Native, Asian American, Asian Indian/Asian Chinese/Asian Filipino/Asian Korean/Asian Vietnamese, other Asian, Hispanic/Latin American, Middle Eastern/North African, Native Hawaiian/other Pacific Islander, White/Caucasian, another race/ethnicity not listed [as an open-ended question], or do not know), preferred language (English or Spanish), and SNAP participation in the past year (yes or no).

We assessed household food security using the USDA 6-Item Food Security Survey which examined the caretakers’ concerns about their household food quantity, quality, and variety in the past month [34]. We categorized food security as very low (five to six affirmative), low (two to four affirmative), or high or marginal (zero to one affirmative) [34]. Compared to the 18-item U.S. Household Food Security Survey, the USDA 6-item Food Security Survey is less burdensome and has a reasonably high specificity, high sensitivity, and minimal bias [39].

### 2.4. Analysis

We conducted descriptive analyses to include frequencies and percentages for categorical variables as well as the median and interquartile range (IQR) for continuous variables of the caretakers’ demographic characteristics (i.e., age, sex, race/ethnicity, and preferred language), household food security, SNAP participation in the past year, indicators of household composition (i.e., household size, number of children ≤ age 10, children ≤ age 5, children ages 6–10, children participating in a school lunch program, and adults), and caretakers’ FV intake.

We analyzed the normality of residuals using the Shapiro–Wilk test which demonstrated violations of the assumption when regressing indicators of household composition on FV daily servings. We categorized FV intake as meeting or not meeting the DGA recommendations as follows: ≥2 servings/day as meeting the fruit intake recommendation, ≥2.5 servings/day as meeting the vegetable intake recommendation, and ≥3.5 servings/day as meeting the FV intake recommendation [8]. Since the NCI FV Screener collects data on serving sizes in addition to cup equivalents, we chose to report serving sizes rather than cup equivalents to reflect the varied definitions of cup equivalents across different types of fruits and vegetables. Reporting serving sizes accounts for varied intake of raw and cooked vegetables, whole fruits, and juice consumption among our population. We conducted log-binomial regression models [40,41] to assess the association between indicators of household composition and adherence to FV recommendations adjusted for the caretakers’ age, sex, race/ethnicity, preferred language, household food security, and SNAP participation in the past year. Despite the relatively moderate sample size, log binomial models were appropriate to analyze the common binary outcome of FV intake [42]. We treated each indicator of household composition as an independent variable and analyzed it in separate multivariate models. To ensure strict model stability, prevent overfitting, and guarantee robust parameter estimation, we employed the *logbin* package in R, utilizing the combinatorial EM (expectation-maximization) algorithm. The convergence tolerance was set to an absolute deviance/parameter change of 1 × 10^−6^ with a maximum iteration threshold of 100 to guarantee stable optimization. Type III effects were evaluated using Wald chi-square tests via the *car* package to assess the independent contribution of each predictor. All models converged successfully and Type III effects are reported in Table A1. We used R to perform all analyses and considered 5% as the significance level.

## 3. Results

### 3.1. Sample Description

A total of 85 caretakers completed the survey. Participants were mainly < age 40 (n = 67, 79%), female (n = 80, 94%), Hispanic (n = 65, 77%), and preferred to speak Spanish (n = 48, 57%) (Table 1). They predominantly resided in households with very low or low food security (n = 77, 91%) and had received SNAP benefits in the past year (n = 68, 80%). The median household size (IQR) was five individuals (3–5), with two (2–3) adults and two (1–3) children ≤ age 10. Children included one (1–2) ≤ age 5, one (0–1) aged 6–10, and one (1–2) participating in a school lunch program.

### 3.2. Caretakers’ FV Intake

Most caretakers did not meet the DGA recommendations for FV intake (n = 54, 63.5%). On average, participants reported consuming 1.13 (0.5–2.21) servings per day of fruits and 1.96 (1.18–2.98) servings per day of vegetables (Table 2). Caretakers who met the FV intake recommendation (n = 42, 49%) reported consuming 3.46 (2.36–5.27) FV servings per day.

### 3.3. Association Between Indicators of Household Composition and Caretakers’ FV Intake

The results of the log-binomial regression models (Table 3) demonstrated that there was a 40–50% greater likelihood of caretakers meeting the DGA vegetable intake recommendation for each additional child in the household (relative risk [RR] 1.44; 95% confidence interval [CI] 1.11–1.86) and each additional child participating in a school lunch program (RR 1.52; 95% CI 1.03–2.24), independently of covariates. Similarly, caretakers who lived with more children (RR 1.36; 95% CI 1.15–1.59), reported living with a higher ratio of younger (≤age 5) to older children (ages 6–10) (RR 1.22; 95% CI 1.07–1.39) or had more children participating in a school lunch program (RR 1.47; 95% CI 1.16–1.85) were more likely to adhere to the FV intake recommendation. Conversely, for each additional adult per child in the household, there was an 18% lower likelihood of meeting the FV intake recommendation (RR 0.82; 95% CI 0.68–0.99).

We found no statistically significant associations between indicators of household composition and the caretakers’ fruit intake. Similarly, there were no significant associations between either ratio variable (adult–children or younger–older children) and the caretakers’ vegetable intake and between household size and caretakers’ fruit, vegetable, and FV intake.

## 4. Discussion

The number of children, children’s age, their participation in a school lunch program, and the number of adults were associated with caretakers’ adherence to the DGA FV intake recommendations among those eligible for the SNAP with a child(ren) in the Bronx, NYC. Our results highlight the importance of further examining the role of indicators of household composition in FV intake and elucidate their possible contribution to disparities of FV intake among urban populations of low income.

When specifying the number of children as an indicator of household composition, we found a positive association with the caretakers’ adherence to the DGA recommendations for vegetable and FV intake. Our results challenged previous studies that found unhealthier dietary patterns among caretakers with children compared to those without [21,22,23,24,25]. Possible explanations for the discordance are that previous studies compared parents versus non-parents, finding that households with biological parent caretakers, especially mothers, reported higher consumption of sugar-sweetened beverages, higher energy, and higher percent total saturated fat than non-parent caretakers (step parents or adoptive, non-biological parents) [21,22,23,24,25]. Many of these studies were also conducted outside of the U.S., providing a different socioeconomic and cultural context, thus not accounting for these influences on food availability and choice in the U.S. While our study participants were homogenous in the sense of having low income and food insecurity, they represent a heterogeneous sample of adults with children, including non-parent caretakers, such as grandparents, relatives, and adoptive parents, thus showing the eating behaviors of all types of caretakers as a whole. Furthermore, the scope of one study limited participants’ eligibility to parents of children ≤ age 5 [21], while our study included a wider age range of ≤age 10, thus broadening the findings of FV consumption.

Concurrently, we found that caretakers living with a greater ratio of younger (≤age 5) to older children (6–10) were more likely to meet the DGA FV intake recommendation, consistent with the implications of multiple studies [27,28,31,32]. This may be due to more younger children in Head Start centers participating in food assistance programs than older children; however, we did not collect information regarding participation in food assistance programs apart from the SNAP. A report focused on child and family characteristics of Head Start centers’ participants across the U.S. showed that 55% of participants participated in the SNAP, 56% participated in the Special Supplemental Nutrition Program for Women, Infants, and Children (WIC), 13% participated in the Temporary Assistance for Needy Families (TANF), and 7% participated in the Supplemental Security Income (SSI) program [43]. Although we did not measure enrollment in programs other than the SNAP, it is possible that households with younger children may be able to purchase and consume more FVs compared to households with older children because of the ability to enroll in additional food assistance programs.

Another possible reason for the negative association between children’s age and caretakers’ FV intake adherence is that multiple studies found that children who are older consume fewer FVs, especially fruits, than their younger counterparts [27,28,31]. One explanation for this decrease in FV intake among older children may be that they have more independence and are more likely to consume foods outside of the home. As a result, caretakers with children who are older may purchase fewer FVs, reducing their availability in the home and subsequently decreasing other household members’ FV intake [44,45]. Our study found no significant association between children’s age and adult caretakers’ fruit intake alone. Most of the participants identified as Hispanic who, according to the latest data, have the highest prevalence (16.4%) of meeting the fruit intake recommendation compared to other ethnicities [9].

We found a positive association between the number of children participating in a school lunch program and the caretakers’ adherence to the DGA recommendations for FV intake. Our result is consistent with the implications of previous studies in the literature reporting that participation in a school lunch program increased food security among families of low income with children by providing nutritional resources for children and consequently creating opportunities to reallocate food to other household members [29,30]. It is important to note that previous studies in the literature investigating enrollment in the National School Lunch Program were large prospective observational studies across the U.S. Given our small sample size and cross-sectional study design, several differences among population, including location, ethnicity, and income level, cannot be accounted for.

Lastly, we found that having a greater adult–children ratio reduced adherence to the DGA FV intake recommendation. Our results contradict the implications of two studies that found healthier dietary habits among adults living with other adults than adults living with children [22,23]. A possible explanation for the discrepancy is that the composition of adults in the household (i.e., spouse/partner or other adults who are not spouse/partner) may play a role in the FV intake of other members in the household [24]. For instance, a study found that men have healthier dietary patterns when living with a spouse compared to another adult who was not a spouse [46]. Our study did not explore the roles of adult caretakers in the household or their employment status. It could be that there were adults living in the household who were employed or receiving food assistance among our population sample, which could increase the ability to purchase and consume FV. In addition, our study did not inquire about the number of adults in the household. We estimated the total number of adults as household members > age 10 by subtracting the number of children ≤ age 10 from the household size. Considering adults as household members > age 10 may include older children and adolescents who may share more similar dietary habits to children than adults. For instance, according to a report about the Healthy Eating Index (HEI) score of children and adolescents, individuals ages 5–10 and ages 11–18 have a similar HEI score ranging from 51 to 55 [32].

Our study has several limitations, one of which is its cross-sectional design that does not allow causal inference and the use of a small sample size of caretakers. Moreover, the use of online surveys may have introduced selection bias towards participants who have smartphones, access to the Internet, and higher possible engagement with institutional programs. To address this bias, we had tablets available during recruitment to allow interested participants to answer the online survey. Our sample size was small and homogenous (i.e., low-income families with young children) and not representative of the heterogenous population in the Bronx in terms of household composition, food security status, and cultural dietary patterns, thereby limiting the generalizability and external validity of the findings. Additionally, an a priori statistical power analysis was not conducted, and the study may be underpowered to detect smaller effects. Given our small sample size relative to the number of covariates adjusted for in the regression models, there is an inherent risk of model overfitting. Consequently, these findings should be interpreted with caution as preliminary and hypothesis-generating rather than definitive. Nevertheless, we were able to successfully recruit and assess a community-based sample of a hard-to-reach population. As secondary analysis of a parent pilot study [47] that utilized a small sample by design due to limited funding, these exploratory findings provide valuable preliminary insights that can inform larger, fully powered future investigations. Lastly, residual confounding may still be possible regardless of statistical adjustment on several covariates. We did not ask about the number of adults in the household, which does not account for children ≥ 10 or adolescents living in the household. Participation in other food assistance programs besides the SNAP or whether the caretakers resided in a household with a single parent were not measured. These variables may contribute valuable information pertaining to household composition.

## 5. Conclusions

Our results challenge the understanding that having fewer children or more adults living in a household increases the likelihood of caretakers’ healthier dietary habits. Conversely, our results align with the findings of the existing literature that a household with more children who are younger or more children participating in a school lunch program is a protective factor to FV intake among caretakers, especially those of Hispanic families eligible for the SNAP. Our results have implications that may benefit not only caretakers with children but also families living in these households, as the diets of caretakers will influence a child’s food choices throughout life. Lastly, our results urge future studies to move beyond children’s presence in the household as the only indicator of household composition when considering caretakers’ FV intake and to support the investigation of social, cultural, and gender dynamics that may influence intrahousehold food distribution and intake.

## Figures and Tables

**Table 1:** Descriptive information of participants in terms of demographic characteristics, household food security, SNAP participation, and indicators of household composition (n = 85).

Demographics		Sample Size (%)	Median (IQR)
**Age (years)**			
	18–29	27 (31.8)	
	30–39	40 (47.1)	
	40 or older	18 (21.2)	
**Gender, Female**		80 (94.1)	
**Race/Ethnicity**		84 (100)	
	African American	14 (16.7)	
	Hispanic	65 (77.4)	
	Multiracial	3 (3.6)	
	Other	2 (2.4)	
**Preferred Language**			
	English	37 (43.5)	
	Spanish	48 (56.5)	
**Food Security**			4 (2–6)
	High or marginal food security (0–1)	8 (9.5)	
	Low food security (2–4)	38 (44.7)	
	Very low food security (5–6)	39 (45.9)	
**SNAP Participation**			
	Received SNAP benefits in the past year	68 (80)	
**Indicators of Household Composition**			
Household Size			5 (3–5)
	Two	9 (10.6)	
	Three	13 (15.3)	
	Four	20 (23.5)	
	Five	28 (32.9)	
	Six or more	15 (17.7)	
Number of Children ≤ Age 10			2 (1–3)
	One	31 (36.9)	
	Two	28 (33.3)	
	Three or more	25 (29.8)	
Number of Children ≤ Age 5			1 (1–2)
	None	7 (8.4)	
	One	54 (65.1)	
	Two or more	22 (26.5)	
Number of Older Children (Age 6–10)			1 (0–1)
	None	38 (45.8)	
	One	32 (38.6)	
	Two or more	13 (15.6)	
Number of Children Participating in a School Lunch Program			1 (1–2)
	One	42 (56)	
	Two	26 (34.7)	
	Three or more	7 (9.3)	
Number of Adults			2 (2–3)
	None	4 (4.8)	
	One	14 (16.7)	
	Two	33 (39.3)	
	Three	15 (17.9)	
	Four or more	18 (21.5)	

Abbreviations: SNAP = Supplemental Nutrition Assistance Program; IQR = interquartile range. Other ethnicities included American Indian/Alaska Native, Asian descent, White, another ethnicity not listed, or unknown.

**Table 2:** Descriptive characteristics of participants adhering to the DGA 2020–2025 recommendations and median intake among participants.

Fruit and Vegetable Intake (Servings/Day)	n (%)	Median (IQR)
Fruits only	31 (36.5)	1.13 (0.5–2.21)
Vegetables only	31 (36.5)	1.96 (1.18–2.98)
Fruits and vegetables	42 (49.4)	3.46 (2.36–5.27)

Abbreviations: DGA, Dietary Guidelines for Americans; IQR, interquartile range.

**Table 3:** Log-binomial regression model of the association between indicators of household composition and caretakers’ adherence to the DGA ^b^ 2020–2025 recommendations for FV ^a^ intake.

Indicators of Household	Fruit Intake			Vegetable Intake			FV Intake		
	RR ^c^	95% CI ^d^	*p*-Value	RR	95% CI	*p*-Value	RR	95% CI	*p*-Value
Household size	1	0.86–1.17	0.997	0.97	0.79–1.18	0.759	0.98	0.85–1.14	0.806
Number of children	1.19	0.95–1.50	0.13	1.44	1.11–1.86	0.005 **	1.36	1.15–1.59	<0.001 ***
Ratio of younger children (<age 5) to older children (age 6–10)	0.98	0.60–1.59	0.925	0.93	0.59–1.46	0.746	1.22	1.07–1.39	0.003 **
Number of children participating in a school lunch program	1.25	0.94–1.66	0.133	1.52	1.03–2.24	0.035 *	1.47	1.16–1.85	0.001 **
Ratio of adults to children	0.84	0.65–1.09	0.191	0.79	0.60–1.05	0.101	0.82	0.68–0.99	0.049 *

^a^ FV = fruit and vegetable. ^b^ DGA = Dietary Guidelines for Americans. ^c^ RR = relative risk. ^d^ CI = confidence interval model adjusted on the caregiver’s sex, race/ethnicity, preferred language, household food security, and SNAP participation in the past year. We treated each indicator of household composition as an independent variable and analyzed it in separate models. * *p*-value < 0.05, ** *p*-value < 0.01, *** *p*-value < 0.001.

## Data Availability

The data that support the findings of our study are available from the corresponding author, K.M., upon reasonable request.

## Abbreviations

The following abbreviations are used in this manuscript:

FV	Fruit and Vegetable
DGA	Dietary Guidelines for Americans
SNAP	Supplemental Nutrition Assistance Program

## Appendix A

**Table A1:** Type III effects of binomial logistic regression models of study variables (n = 85).

Model	Wald Chi-Square ^a^	df ^b^	*p*-Value
Fruit Intake			
Household size	0.398	1	0.528
Children ≤ age 10 receiving school lunch	0.395	1	0.530
Ratio of adults to children	0.111	1	0.739
Ratio of younger to older children	0.295	1	0.587
Children ≤ age 10 living in the household	0.009	1	0.924
Vegetable Intake			
Household size	0.029	1	0.865
Children ≤ age 10 receiving school lunch	2.50	1	0.114
Ratio of adults to children	2.169	1	0.141
Ratio of younger to older children	0.088	1	0.767
Children ≤ age 10 living in the household	5.17	1	0.023
Fruit and Vegetable Intake			
Household size	0.541	1	0.462
Children ≤ age 10 receiving school lunch	4.566	1	0.033
Ratio of adults to children	3.110	1	0.078
Ratio of younger to older children	0.000	1	0.996
Children ≤ age 10 living in the household	4.515	1	0.034

^a^ Chi-square; ^b^ degrees of freedom.
